# Enhancing methane oxidation in a bioelectrochemical membrane reactor using a soluble electron mediator

**DOI:** 10.1186/s13068-020-01808-7

**Published:** 2020-10-16

**Authors:** Xueqin Zhang, Hesamoddin Rabiee, Joshua Frank, Chen Cai, Terra Stark, Bernardino Virdis, Zhiguo Yuan, Shihu Hu

**Affiliations:** 1 Advanced Water Management Centre, Faculty of Engineering, Architecture and Information Technology, The University of Queensland, Brisbane, 4072 Australia; 2 Australian Institute for Bioengineering and Nanotechnology, The University of Queensland, Brisbane, 4072 Australia; 3 Queensland Node of Metabolomics Australia, The University of Queensland, Brisbane, 4072 Australia

**Keywords:** Bioelectrochemical membrane reactor, Redox mediator, Bioelectrochemical methane oxidation, ANME, Ferricyanide

## Abstract

**Background:**

Bioelectrochemical methane oxidation catalysed by anaerobic methanotrophic archaea (ANME) is constrained by limited methane bioavailability as well as by slow kinetics of extracellular electron transfer (EET) of ANME. In this study, we tested a combination of two strategies to improve the performance of methane-driven bioelectrochemical systems that includes (1) the use of hollow fibre membranes (HFMs) for efficient methane delivery to the ANME organisms and (2) the amendment of ferricyanide, an effective soluble redox mediator, to the liquid medium to enable electrochemical bridging between the ANME organisms and the anode, as well as to promote EET kinetics of ANME.

**Results:**

The combined use of HFMs and the soluble mediator increased the performance of ANME-based bioelectrochemical methane oxidation, enabling the delivery of up to 196 mA m^−2^, thereby outperforming the control system by 244 times when HFMs were pressurized at 1.6 bar.

**Conclusions:**

Improving methane delivery and EET are critical to enhance the performance of bioelectrochemical methane oxidation. This work demonstrates that by process engineering optimization, energy recovery from methane through its direct oxidation at relevant rates is feasible.

## Background

Methane (CH_4_) is an important energy resource that has attracted increasing attention due to rapidly growing energy demand. CH_4_ resource is abundant with two sources: fossil natural gas with growing proven reserves, and biogas with renewable availability [1, 2]. Thus CH_4_ provides us a long-term energy sustainability. Moreover, CH_4_ is also recognized as a potent greenhouse gas (GHG), with its global-warming potential 28–34 times that of carbon dioxide over a 100-year time frame [3]. It is therefore desirable for direct CH_4_ utilization or upgrading on-site to minimize its global-warming potential. Both factors sparked interests on developing effective CH_4_-based technologies to produce energy whilst mitigating its adverse impact on climate change [4, 5].

While direct CH_4_ combustion in gas turbines has been a widely implemented strategy for energy recovery from CH_4_, this approach is constrained by the inherently low volumetric energy density for transportation sector and low energy efficiency for electricity generation [6]. Proposed alternatives for CH_4_ utilization include direct CH_4_ conversion into electricity, for example in solid fuel cells (SOCFs) [5, 7], and CH_4_ conversion into liquid fuels (e.g., hydrocarbons) through thermochemical processing [5, 6]. The approach of converting CH_4_ into electricity in SOCFs is technically challenging due to the high-temperatures required and the instability of the catalysts [5, 8]. Conversely, gas-to-liquid (GTL) technologies such as the well-established Fischer–Tropsch (FT) process, while attractive for its capability to convert CH_4_ into valuable and energy-dense liquid chemicals and fuels including methanol, longer chain hydrocarbons, olefins, and gasoline, is challenged by high technical complexity as well as numerous heat and pressure changes [6].

Benefited from increasing awareness of the feasibility of C–H bond activation by biocatalysis, biotechnologies are recently proposed to have good opportunities to support bioconversion of CH_4_ into electricity or liquid chemicals with virtues of mild operational conditions and technical simplicity over SOCFs and FT process [9, 10]. In this regard, microbial electrochemical technologies have recently been suggested as an effective method for the conversion of CH_4_ directly into electricity or indirectly into value-added chemicals and fuels through electrochemical conversions catalysed by whole living microbial cells [5]. Bioelectrochemical CH_4_ transformation into versatile products can overcome its limitation of low volumetric energy density. Bioelectrochemical conversion of CH_4_ to electricity, compared to direct CH_4_ combustion in turbine, can achieve a higher efficiency since it avoids the Carnot cycle and produces primarily electricity instead of heat [11].

While the concept of a CH_4_-fueled bioelectrochemical systems (BESs) has been speculated ever since the birth of microbial electrochemistry as a research field [12], the difficulty in finding a suitable biocatalyst displaying abilities of both anaerobic CH_4_ activation and extracellular electron transfer (EET) has limited the number of successful reports to a handful [5, 13]. Indeed, the demonstration of direct EET as the necessary mechanism to enable CH_4_ oxidation coupled with sulfate reduction [14, 15], or with insoluble iron and manganese oxides [16, 17] by some lineages of anaerobic methanotrophic archaea (ANME) suggested that ANME might have the ability to respire on solid electrode surfaces. Recently it has been demonstrated that ANME-2d can couple anaerobic oxidation of methane (AOM) to electrode reduction [18].

While the implications of direct electricity production from CH_4_ oxidation in BESs at ambient temperature is feasible, the technology is far from large-scale implementations due to the low current densities currently achievable [19], which are normally orders of magnitude lower than typically achieved by their bacterial electroactive counter parts, e.g., *Geobacter* [20]. The typically low rates of AOM catalysed by ANME and coupled with electrode reduction in BESs are probably the result of two rate-limiting processes, including (1) poor mass transfer rate and solubility of CH_4_ in the cultivation media, which limits microbial accessibility to CH_4_; (2) electron transfer from the microbial cells to the final external electron acceptor (i.e. the anode electrode).

As a possible solution to the poor CH_4_ mass transfer issue, our group has previously proposed the use of hollow fibre membranes (HFMs) to supply CH_4_ gas in membrane biofilm reactors (MBfRs) driving denitrification, resulting in an increase in the denitrification performance by 20–30 times relative to a control [21, 22]. This significant improvement can be understood by considering that in MBfRs, methanotrophic biofilm colonize and grow on the outer surface of the membranes, having therefore direct access to CH_4_ at supersaturation levels. This strategy, while effective when coupling CH_4_ oxidation with the reduction of a soluble electron acceptor such as nitrate, cannot directly be implemented in BESs where the electron acceptor is a solid-state electrode. To be effective, it is necessary to establish an electrical connection between the methanotrophic biomass on HFMs and the electrode.

In this work, we tested the hypothesis according to which a soluble redox mediator can be used to shuttle electrons between methanotrophic organisms and the anode electrode, thereby “electrochemically bridging” the HFMs (and the biofilm within) and the electrode without the requirement to physically grow the methanotrophic organisms on the surface of the electron-accepting anode. We inoculated our systems with an enriched culture of ANME organisms and amended potassium ferricyanide as the redox mediator. The current knowledge of ANME metabolism when using insoluble electron acceptors points at the involvement of *c*-type cytochromes in extracellular electron transfer [14, 16, 23, 24]. Therefore, we used a redox mediator with a mid-point potential sufficiently high to effectively extract electrons from *c*-type cytochromes (*E*^0′^ for ferricyanide is ca. + 416 mV vs the Standard Hydrogen Electrode at pH 7 [25], while the *E*^0′^ for *c*-type cytochromes is reported at ca. − 200 to 300 mV [26]), as well as for its high electron transfer kinetics at graphite electrodes [27]. We then characterised the bioelectrochemical membrane reactor (BEMR) system in terms of current output and community dynamics and compared the performance with a Control-BES where neither the HFMs nor the mediator was used.

## Results

### ANME couple methane oxidation with ferricyanide reduction

**Fig. 1:**
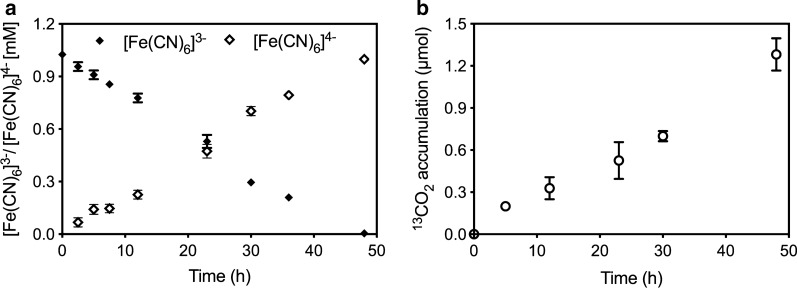
Ferricyanide reduction by ANME-dominated culture in batch incubations: **a** change in ferricyanide and ferrocyanide concentrations (mM) and **b** accumulation of ^13^CO_2_. ^13^CH_4_ was fed in the headspace of incubations at a partial pressure of 0.67 atm

To validate if ferricyanide can mediate electron transfer from ANME to the electrode, the ability of the ANME-dominated culture to reduce ferricyanide with CH_4_ as electron donor was firstly investigated. In incubations containing microbial inoculation and CH_4_ amendment, the characteristic dark yellow colour of ferricyanide in oxidised form gradually faded, and completely disappeared after 48 h (Additional file 1: Fig. S1), yielding a clear uncoloured solution, indicating the progressive reduction of ferricyanide and its conversion into ferrocyanide (Fig. 1a). By comparison, abiotic control (without inoculum) displayed no visible colour change during the incubation, further proving that the ferricyanide reduction observed in the flasks amended with ANME organisms was of a biological nature. Further, the progressive accumulation of ^13^CO_2_ indicated ^13^CH_4_ oxidation (Fig. 1b), confirming the presence of biological conversions associated with ferricyanide reduction.

### Colonization of methanotrophic biofilm on HFMs

**Fig. 2:**
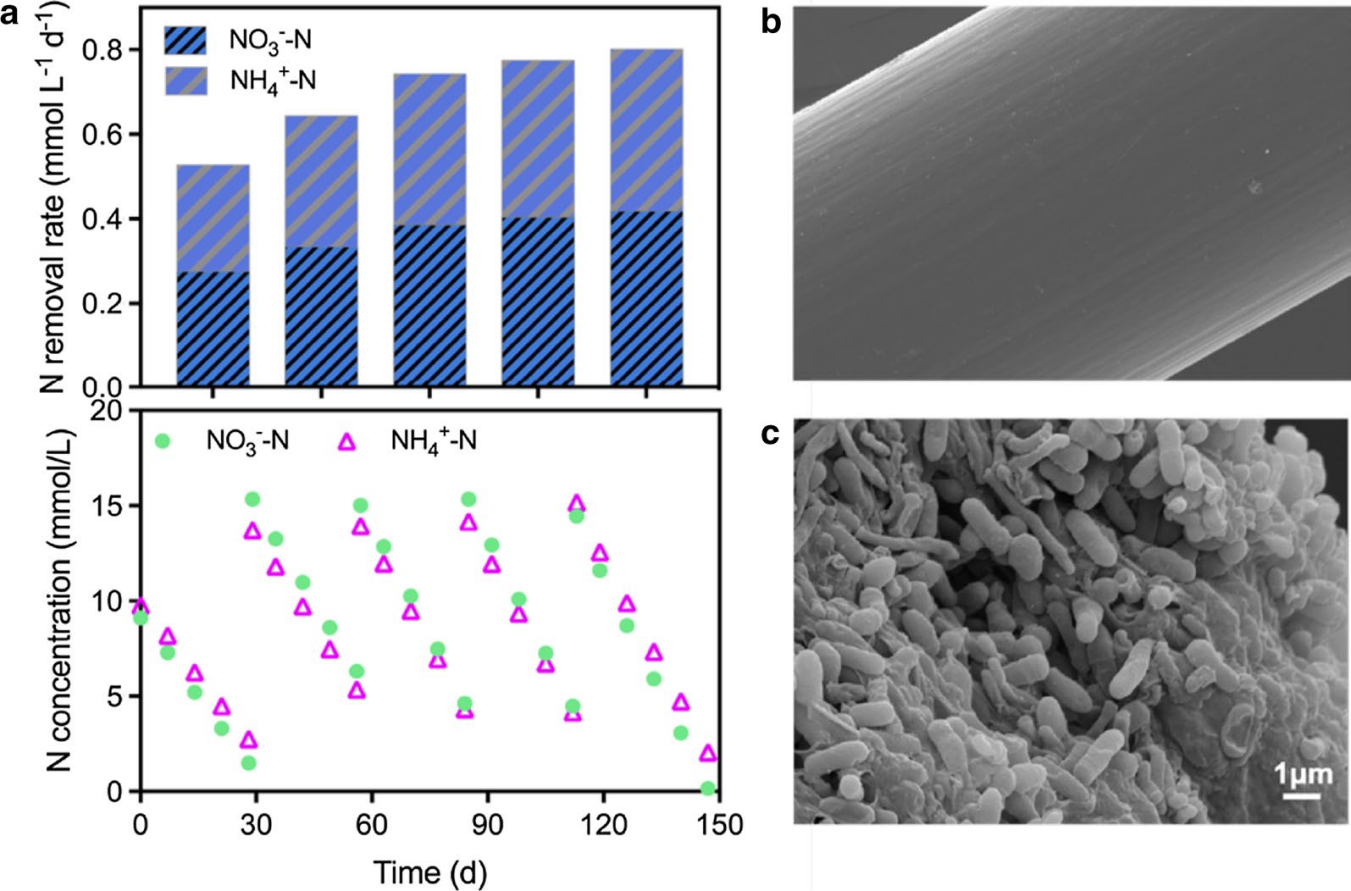
Progressive colonization of methanotrophic biofilm on HFMs. **a** Nitrate and ammonium concentration variations and removal rates with pulse nitrogen nutrition feeding during operational stage 1 in the BEMR. **b** Scanning electron microscope (SEM) image of hollow fibres before microbial colonization on day 0. **c** SEM image of methanotrophic biofilm on hollow fibres at the end of stage 1 (day 147) in the BEMR

Bubble-less gas exchange HFMs have been shown as effective for delivering gaseous substrate of CH_4_ because CH_4_ can be delivered directly from the inside of HFMs to a biofilm attached on the outer surface of HFMs [21, 28]. Thus, colonization of a CH_4_-oxidizing biofilm on HFMs is the prerequisite for a highly efficient CH_4_ utilization system. As the methanotrophic biomass used here as inoculum was adapted to nitrate as the electron acceptor, we fed nitrate (and ammonium) during Stage 1 of operation to stimulate colonization of methanotrophic biofilm on HFMs. Figure 2a reports the profiles of nitrate and ammonium concentration (and removal rates) over time during multiple batch feeding tests. Nitrate was removed with rates increased over time throughout the operational course of the first 147 days, indicating the gradual colonization of methanotrophic biofilm on the surface of HFMs and confirming our previous observations [21, 29]. Ammonium removal was also observed since the anammox microorganisms contained in the inoculum oxidized ammonium using the nitrite produced by ANME archaea from nitrate reduction, which has been described before [23]. The attachment of methanotrophic biofilm on the HFMs could be also verified by SEM imaging. At the end of operational Stage 1, a dense biofilm morphology can be observed on the outer hollow fibres, a clear indication of the successful colonization (Fig. 2b,c).

### Enhanced performance of bioelectrochemical CH_4_ oxidation in the BEMR

**Fig. 3:**
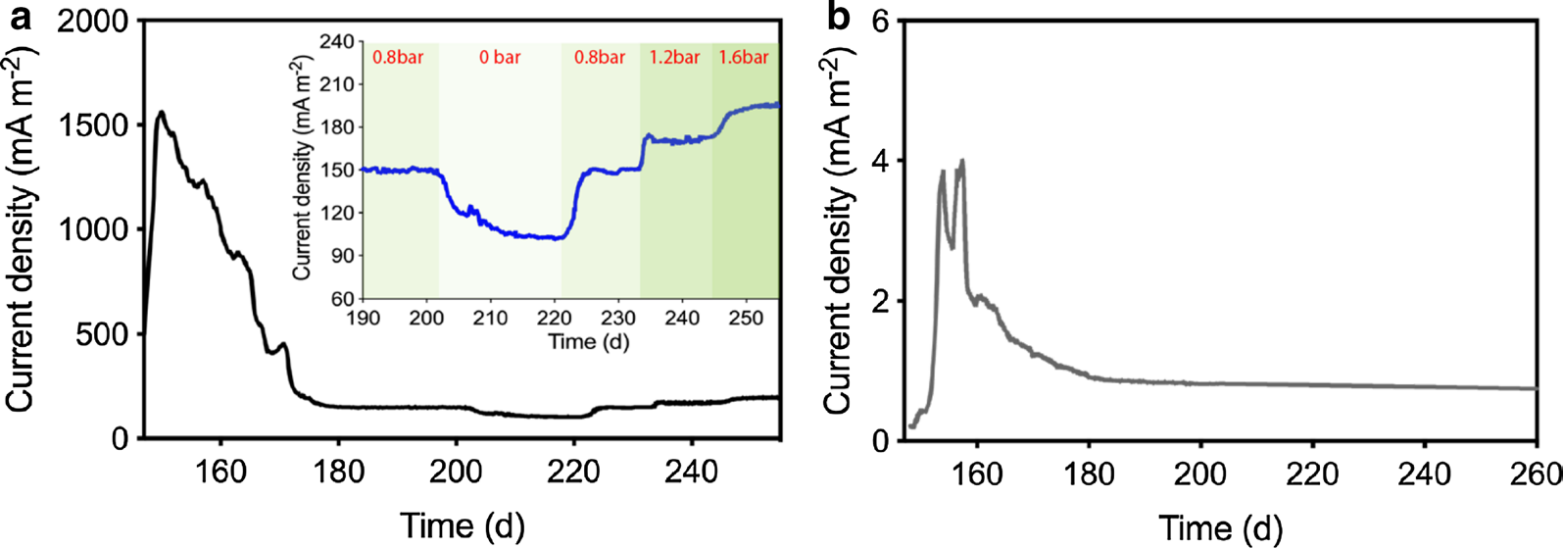
Characteristic current *versus* time traces resulting from bioelectrochemical CH_4_ oxidation. **a** In the BEMR at different CH_4_ pressures; **b** in the Control-BES

Figure 3 shows CH_4_-dependent bioelectrochemical current output by means of chronoamperometry in the BEMR (Fig. 3a) and Control-BES (Fig. 3b). The current versus time traces in both systems display initial peaks and maxima in the first 30 days. Coulombic efficiency estimation showed that only 54.3% of electron recovery can be obtained against the assumption that the current output in this period was solely driven by AOM (Additional file 1: Table S1), which suggests that there was some other more oxidized carbonaceous substances contributed to the current output. This could be ascribed to the oxidation of intracellular storage substances present in methanotrophic biomass providing additional reducing equivalents, as previously discussed [30, 31]. Afterwards, the current in the BEMR stabilized at around 149 mA m^–2^ when the HFMs was pressurized at 0.8 bar. It dropped to approximately 102 mA m^−2^ as soon as HFMs was depressurized to 0 bar (Fig. 3a, insert). In this scenario, the CH_4_ supply pressure was the same to the Control-BES, while the current output in the BEMR was still about 126 times higher than the stabilized current (0.8 mA m^–2^) in the Control-BES. Once the HFMs was re-pressurized at 0.8 bar, the current in the BEMR was quickly recovered to the level of approximately 102 mA m^−2^, and it was further increased to around 170 mA m^−2^ and 196 mA m^−2^ when the pressure applied to HFMs was increased to 1.2 bar and 1.6 bar, respectively.

Regarding the control experiment for confirming the contribution of ferricyanide to increased current output, ferricyanide was gradually removed or replenished to the anode chamber by a manner of medium replacement through continuous pumping. Along with the rapid decline of ferricyanide concentration, the current quickly dropped to around 0.6 mA m^−2^; as soon as ferricyanide was fed back to the anode chamber, the current rapidly recovered before it plateaued at the original level (Additional file 1: Fig. S3).

The enhancement of AOM in the BEMR was further evidenced by significant CO_2_ accumulation observed (Additional file 1: Fig. S4). The calculated coulombic efficiency of BEMR was 96.5 ± 4.9% during the stabilized current period (Day 183 to 255) (Additional file 1: Table S1), which suggests that electrons generated from CH_4_ oxidation can be almost entirely recovered as current output. In comparison, limited CO_2_ production was observed in the Control-BES (Additional file 1: Fig. S4), which was in line with the low current observation (Fig. 3b).

### Electrochemical characterization by cyclic voltammetry

**Fig. 4:**
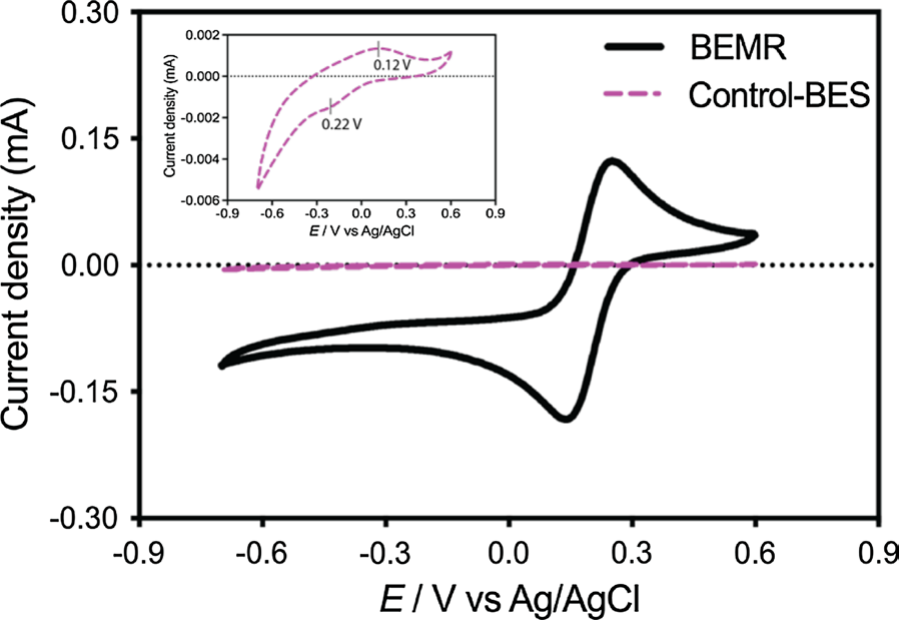
Cyclic voltammetries (CVs) recorded in the BEMR and Control-BES (insert) at the scan rate of 1 mV s^−1^

To understand how ferricyanide enhanced bioelectrochemical oxidation, CVs were recorded and compared in the BEMR and Control-BES (Fig. 4). The voltametric response of the Control-BES (insert in Fig. 4) showed a redox couple centred at + 0.17 V, which could be associated with electroactive biofilm on the graphite anode in the Control-BES (as proven by SEM imaging, Additional file 1: Fig. S5). By contrast, the voltammogram of BEMR revealed a reversible redox process featured by ferri- ferrocyanide redox couple. It confirmed the active role of ferricyanide as an effective mediator in the BEMR. The observed higher voltametric current density on the CV profile of the BEMR indicated the excellent electron transfer kinetics of the mediator involved at graphite electrodes, which could facilitate the electron transfer from the microbial cells to the anode ultimately for the enhanced performance of bioelectrochemical oxidation in the BEMR.

### Microbial community dynamics

**Fig. 5:**
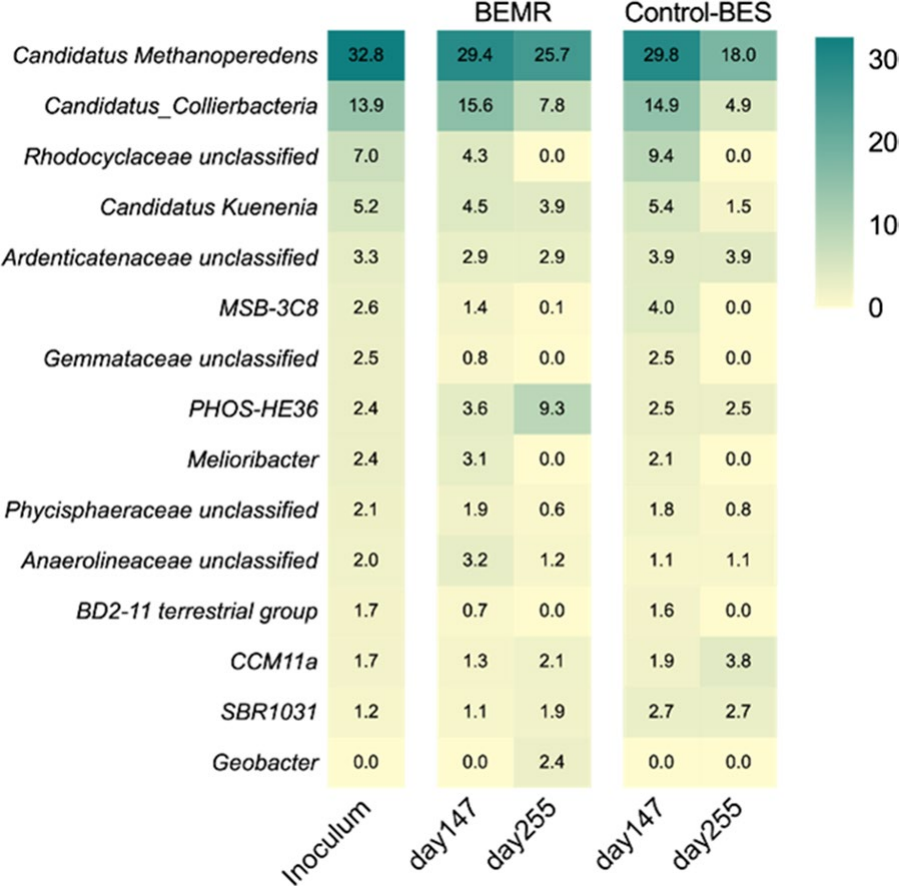
Heat map showing dynamic of microbial community (at genus level) at different operational stages. Day 147 and day 255 represent microbial samples at the end of nitrate-driven stage and polarized electrode-driven stage, respectively. Genera with an abundance of ≥ 1% in at least one sample are presented

To understand biological processes for bioelectrochemical AOM, 16S rRNA gene amplicon sequencing was undertaken to characterize microbial community dynamics both in the BEMR and the Control-BES at different operational stages (Fig. 5).

The methanotrophic community used as the inoculum for both bioelectrochemical systems described in this work was previously enriched in the presence of nitrate as the final electron acceptor. Accordingly, it contained the ANME archaea *Candidatus Methanoperedens* (32.8%) and its syntrophic partner anammox bacterium *Candidatus Kuenenia* (5.2%) [23]. Other bacteria, such as *Candidatus Collierbacteria* (13.9%) and Rhodocyclaceae (7.0%), also coexist in the community with high percentage, however their roles are yet to be confirmed [23]. At the end of operational Stage 1, the 16S rRNA gene profiling results in the BEMR was in line with the inoculum, which suggested the colonization of methanotrophic biofilms on HFMs. When nitrate was depleted and the BEMR was operated with polarized electrode as the electron acceptor, ANME organism *Ca. Methanoperedens* still dominated the microbial community (25.7%) at the end of operational Stage 2 in the BEMR. Meanwhile, no other methane-oxidizing microbes were found to occur in the community, revealing that *Ca. Methanoperedens* was the only candidate being responsible for CH_4_ activation. Interestingly, organisms belonging to the genus *Geobacter* were also observed in the community enriched in BEMR, reaching a relative abundance of 2.4%, which could be stimulated by readily available intermediates (such as acetate) released by intracellular storage substances of methanotrophic biomass [30, 31]. Regarding the Control-BES, the microbial community was also consistent with the inoculum when nitrate was fed in the first 147 days. When nitrate was replaced by polarized electrode as electron acceptor, *Ca. Methanoperedens* also dominated the community without any other CH_4_- or EET-associated microorganisms evolved. Thus *Ca. Methanoperedens* was expected to catalyse bioelectrochemical CH_4_ oxidation in the Control-BES. But compared with the BEMR, a more significant drop (from 29.8% to 18.0%) of *Ca. Methanoperedens* population was observed, which was perhaps related to more sluggish EET kinetic in the Control-BES (Fig. 3).

## Discussion

In the current study, the current output was achieved in a BES (Control-BES) catalysed by an ANME-dominated methanotrophic consortium (Fig. 3b). Microbial community analysis indicated that ANME in the consortium likely implement essential physiological processes independently, including CH_4_ activation and EET to electrode (Fig. 5). However, the current output performance achieved in the Control-BES is extremely poor, which could be mainly attributed to two possible rate-limiting aspects: (i) electron production from CH_4_ that is limited by low CH_4_ bioavailability due to low solubility of CH_4_ in the solution and (ii) electron transfer from ANME to electrode constrained by poor EET ability of ANME.

**Fig. 6:**
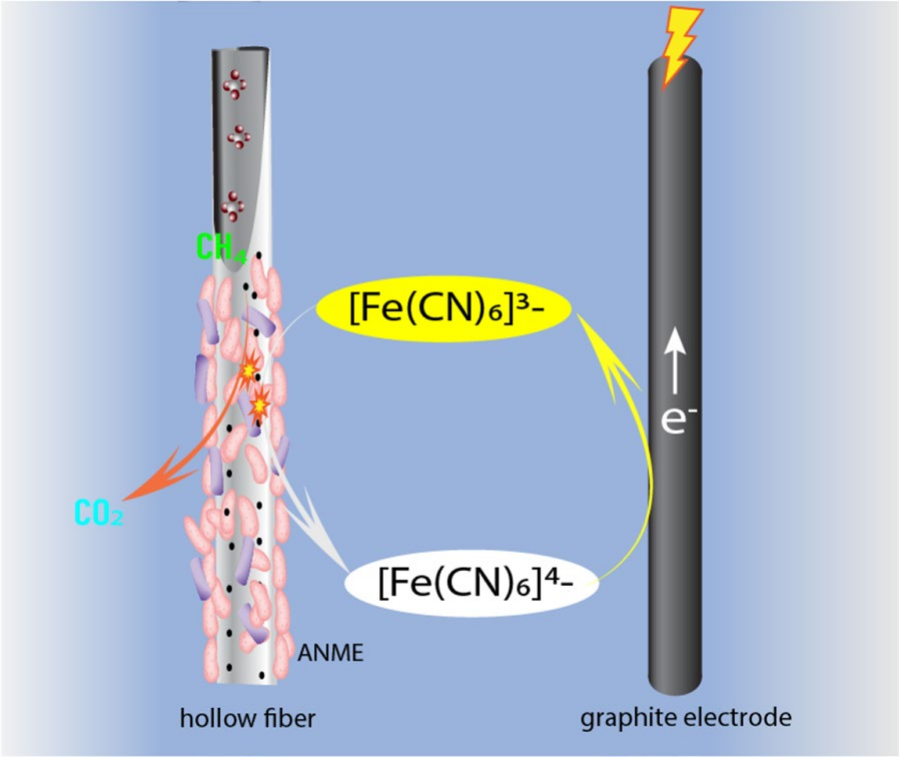
Schematic diagram of working principle in BEMR

For the first aspect, it was proposed to be overcome by an engineering approach in terms of CH_4_ delivery by HFMs [21, 32]. However, the principle cannot be directly applied into BESs, since commercially available HFMs that are made of nonconductive polymer cannot electrochemically interact with the anode to transfer electrons to outside circuit of BESs. In this study, this issue was addressed by amending soluble mediator of ferricyanide in the BEMR. Ferricyanide accepts electrons extracellularly, which means only EET-capable microbes catalyse ferricyanide reduction. The genetic information of ANME reveals that it encodes numerous multihaeme *c*-type cytochromes [23], indicating its EET capability. Actually, a similar ANME species (also belonging to the genus of *Ca. Methanoperedens*) has been proved to be able to reduce other iron species (i.e. ferric citrate, ferrihydrite) [16], which further consolidate the EET physiology of ANME in the inoculum. As no other CH_4_-oxidizing microbes appeared in the enriched methanotrophic culture (Fig. 5), the immediate onset of ferricyanide reduction being correlated to CH_4_ oxidation in batch incubations suggested ANME in the methanotrophic consortium is able to reduce ferricyanide (Fig. 1). These results demonstrated that ferricyanide can be used as an effective mediator to electrochemically bridge the HFMs (and the biofilm within) and the electrode. As shown in Fig. 6, methanotrophic biofilms colonized on the outer surface of HFMs oxidized CH_4_ diffused from inside of hollow fibres with soluble ferricyanide in the solution as the electron acceptor. Ferricyanide capturing electron from CH_4_ oxidation was reduced to ferrocyanide, which can be diffused to physically separated electrode surface and be oxidized to ferricyanide by the polarized electrode. The dynamic redox cycles of ferri/ferrocyanide enabled electrochemical bridging between HFMs (i.e. the site of CH_4_ oxidation) to the electrode (i.e. the site of final electron transfer), achieving sustainable bioelectrochemical CH_4_ oxidation on HFMs. This can be evidenced by the dependence of current on ferricyanide concentration (Additional file 1: Fig. S3). When ferricyanide was removed from the anode, the current dropped to a level matching that of the Control-BES, which revealed the indispensable role of the mediator on bridging electron transfer from the methanotrophic biofilm on HFMs to anode. The CH_4_ bioavailability limitation was overcome with the shuttling role of ferricyanide, as current output performance was accordingly enhanced with pressure increase and it almost doubled when the pressure was increased from 0 to 1.6 bar (Fig. 3).

Regarding the second limitation aspect, electron transfer from ANME to electrode could be constrained by poor EET ability of ANME. Microbial community analysis indicated that ANME likely activated CH_4_ and transferred electrons to the electrode independently (Fig. 5). However, the low current output in the Control-BES (Fig. 3) indicated that ANME seemed to be a weak electricigen in comparison to strong counterparts such as *Geobacter* [33]*.* Use of mediators is suggested as an effective method to improve the EET kinetics of weak electricigen [33], which has been intensively confirmed to enhance performance of BESs with different strains as biocatalysts [34, 35]. This is proved to be applicable to ANME as well in the current study. When ferricyanide was applied as exogenous mediator in our current study, bioelectrochemical CH_4_ oxidation performance in the BEMR was enhanced dramatically by 126 times compared to that in the Control-BES when both were operated with ambient CH_4_ pressure (Fig. 3). Taken together, soluble mediator of ferricyanide enhances bioelectrochemical CH_4_ oxidation in the BEMR not only through directly facilitating EET of ANME, but also through indirectly increasing CH_4_ bioavailability by enabling application of HFMs for CH_4_ delivery. With HFMs pressurized (at 1.6 bar) to delivery CH_4_ substrate, the BEMR outperformed the Control-BES for approximately 244 times at CH_4_-based current output.

To date, multiple strategies have been reported to effectively enhance performance of CH_4_-powered BESs. These strategies, including genetic manipulation to increase methyl-coenzyme M reductase (MCR) expression for faster CH_4_ activation [13], as well as system optimization of configurating gas-diffusion electrode (GDE) to increase CH_4_ bioavailability [19], have been demonstrated to enhance performance by orders of magnitude in comparison to their counterparts without any manipulation on biocatalysts or BES configuration (Additional file 1: Table S2). The engineering approach presented in this study also shows impressive performance enhancement over these unmanipulated systems, although the current density achieved here is still inferior to some manipulated CH_4_-powered BESs reported to date (Additional file 1: Table S2). However, it should be pointed out the aim of this study is to validate the feasibility of a newly raised engineering strategy, while the strategy itself has not been optimized to its best conditions. The highest performance of CH_4_-powered BESs reported to date was indeed achieved by further system optimization on the basis of a convinced biological manipulation [13, 36]. Hence, system optimization is anticipated to further enhance the performance of bioelectrochemical CH_4_ oxidation based on the engineering strategy in the current study. One optimization aspect is to identify the optimal mediator species and concentration to obtain the faster mediated interaction between ANME and electrodes. Another optimization aspect is to further amplify the surface area of HFMs. Compared to the strategy of using GDE to increase CH_4_ bioavailability [19], application of HFMs features the availability of higher membrane pack intensity for the higher surface to volume ratio, which could potentially enable incredible CH_4_ diffusion and biomass incorporation as biofilm on HFMs.

Meanwhile, different methanotrophic consortia with different EET pathways were identified in different studies, which could also be associated with performance variations in different CH_4_-powdered BESs. Specifically, CH_4_ activation is all proposed to be activated by reverse methanogenesis via MCR in different methanotrophic consortia, while EET strategy varied depending on the MCR hosts (Additional file 1: Table S2). An intermediate-dependent EET mechanism has been proposed in different methanotrophic consortia [13, 19], within which CH_4_ activator interact with other electroactive bacteria (i.e. *Geobacter*) by diffusible intermediates, and final electron exchange with electrode are performed by the strong electricigen of *Geobacter* rather than CH_4_ activators themselves. The higher electroactivity of *Geobacter* enables more efficient electrical interaction between methanotrophic consortia and electrodes [33], which could also be the reason for the superior AOM performance in BESs identified with the intermediate-dependent EET mechanism [13, 19]. Nevertheless, compared with the direct interaction between methanotrophs and electrode in our study, the diffusion-dependent EET strategy in methanotrophic consortia is perhaps vulnerable to intermediate loss and environmental chemical fluctuations [14], which can affect energy efficiency for CH_4_ transformation. Hence, a more comprehensive investigation is required to know the importance of physiology in terms of EET strategy in CH_4_-based BESs. Together, our study provides an engineering strategy to enhance CH_4_ oxidation towards CH_4_-based bioelectrochemical technologies, and it also inspires us with architecture design for CH_4_-based BESs from both engineering and biocatalytic consortia perspectives. Due to the high redox potential of ferricyanide, although it is suitable for application in electrode-poised BESs for upgrading CH_4_ to chemicals or fuels, it is less favourable when compared to other mediators for application in a fuel cell mode for direct electricity generation. Further studies are warranted to reveal different fit-for-purpose mediators in different CH_4_-fuelled BESs. By utilizing suitable mediators, practical application of CH_4_ transformation for direct electricity generation or for fuel and chemicals production could be promoted with enhanced performance.

## Conclusions

This study reports the application of redox mediator ferricyanide in a bioelectrochemical membrane reactor (BEMR) to enhance the catalysis of methane oxidation by an ANME-dominated methanotrophic consortium using an electrode as electron acceptor. On the one hand, the diffusible mediator bridges the distancing EET from methanotrophic biofilm on the non-conductive hollow fibres to physically separated anode, ultimately enabling membrane-based CH_4_ delivery for enhanced CH_4_ bioavailability. On the other hand, the mediator enhances EET kinetics of ANME directly by introducing the mediator-directed EET strategy. With hollow fibres pressurized at 1.6 bar to deliver CH_4_, the BEMR amended with mediator outperformed the Control-BES for approximately 244 times at CH_4_-based current output. Hence, this work provides an engineering strategy to enhance bioelectrochemical CH_4_ oxidation with ANME as the biocatalyst.

## Methods

### Batch incubations to test AOM coupled to ferricyanide reduction

Ferricyanide reduction theoretically can be coupled with CH_4_ oxidation to CO_2_, which can be described by the following equation [37]. The Gibbs energy value is calculated based on standard conditions (pH 7, 298 K, 1 atm).

$$ {\text{CH}}_{4} + \, 8\left[ {{\text{Fe}}\left( {{\text{CN}}} \right)_{6} } \right]^{3 - } + \, 2{\text{ H}}_{2} {\text{O }} \to \, 8\left[ {{\text{Fe}}\left( {{\text{CN}}} \right)_{6} } \right]^{4 - } + {\text{ CO}}_{2} + \, 8{\text{H}}^{ + } ;\quad \Delta {\text{G }} = \, - 463{\text{ kJ/mol}} $$

Before operating the bioelectrochemical systems described above to verify whether ferricyanide can be used as a soluble mediator to shuttle electrons between the HFMs-supported biofilm and the anode, we first assessed whether our methanotrophic culture was in fact able to catalyse ferricyanide reduction during CH_4_ oxidation, hence providing proof that the redox mediator was able to extract electrons from AOM.

Incubations were performed in 35 mL serum bottles (Sigma, USA). Methanotrophic biomass dominated by ANME archaea and anammox bacterium was used as the inoculum [23]. Biomass collected from parent reactor was centrifuged at 6000 rpm for 15 min. The resulting biomass pellet was resuspended in fresh medium without nitrate (i.e. the basic medium used in the parent reactor, which composition was reported previously [38]). The procedure was repeated three times to eliminate any residual nitrate. Finally, the biomass was resuspended into triple volume of fresh medium (to yield a volatile suspended solids (VSS) concentration of *ca*. 0.25 g/L). K_3_[Fe(CN)_6_] was added into the culture resuspension at a concentration of 1 mM. The biomass was then distributed into 20 mL aliquots to each serum bottle, after which the bottles were sealed with butyl rubber stoppers and aluminium crimp caps. 10 mL ^13^CH_4_ (> 99.99% 13C; Sigma-Aldrich) was injected into the headspace each vail to provide the electron donor. All these operations were processed inside an anaerobic chamber (Coy Laboratory Products Inc., USA). Incubations were performed in triplicates, at room temperature (24 ± 1 °C), and in the dark. 1.0-mL liquid samples were routinely taken from the flasks and analysed to determine K_3_[Fe(CN)_6_] reduction and ^13^CO_2_ accumulation (see analytical methods below). 0.5 mL of liquid samples were filtrated for K_3_[Fe(CN)_6_] and K_4_[Fe(CN)_6_] quantification immediately; another 0.5 mL of liquid samples were taken and injected into 3 mL Exetainer vials (Labco, UK) for ^13^CO_2_ measurement.

### BEMR configuration and operation

**Fig. 7:**
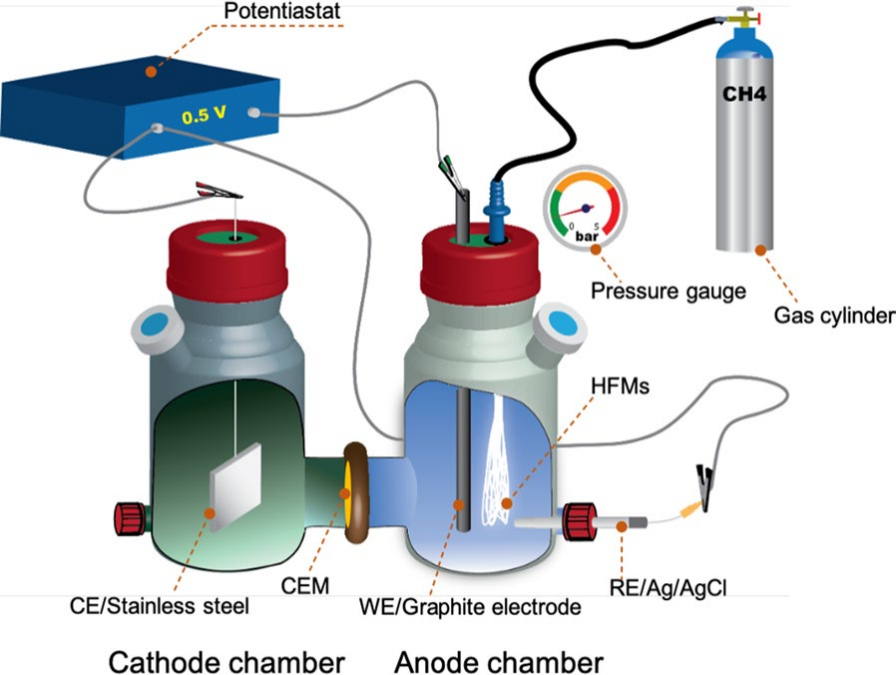
The schematic diagram of configuration and operation of BEMR. *WE* working electrode, *CE* counter electrode, *RE* reference electrode

The schematic diagram of BEMR configuration and operation are illustrated in Fig. 7. It was constructed on the basis of a H-type electrochemical reactor configuration, which was assembled by using two commercially available glass bottles (Wenoote, China), each somewhat modified to accommodate various sampling ports and two flanges to allow clamping the bottles together. A piece of cation-exchange membrane (CMI-700, Membrane International Inc., USA) was cut into the size fitting the connection flange. It was then sandwiched between two pieces of pierced rubber gaskets, which was assembled between two bottles to separate them into two chambers (anode and cathode chamber). Each chamber had a total (i.e. empty volume) volume of 210 mL, and the working volume (i.e. liquid volume) was 160 mL. The top port of each bottle was sealed by a rubber stopper (Cole-Parmer, USA), kept in place by a plastic screw cap. Provision of CH_4_ to the anode chamber was achieved using a bundle of HFMs consisting of 300 pieces of hollow fibres (TM 830Y K200 nonporous polypropylene hollow fibre, Teijin Fibers, Ltd., Japan) each with length of 20 cm and an inner/outer fibre diameter of 50/200 μm. The HFM bundle was folded from the middle, then the two ends were jointed as such that all membrane sections were aligned. The end the HFM bundle was packed into a tubing fitting and then cemented together with epoxy glue. The fitting was pierced through the stopper, leaving a protruded part for tubing connection. Above assembly resulted into an effective membrane surface area (under anodic electrolyte) of 301.4 cm^2^. The end of the HFM was connected to a CH_4_ gas cylinder through a gas-impermeable hose. CH_4_ was fed to the anodic chamber by pressurizing the lumen of the HFM using a regulator, thus CH_4_ was forced to penetrate through the wall of the HFM. Next to the HFM bundle, a graphite rod (6.0 mm in diameter, Graphite Sales Inc., US) pierced through the rubber stopper with 7 cm immersed into the solution, was used as the anode electrode. In the cathode chamber, a piece of stainless steel fibre felt (5 cm × 2.5 cm × 1 mm, Lier Filter Ltd, Henan, China) was used as cathode electrode. External electrical continuity was guaranteed by connecting the cathode a titanium wire, which was then pierced through the rubber stopper. To ensure gas-tightness of each chamber, epoxy glue was applied onto every holes on the rubber stoppers where electrodes or fittings were installed.

The BEMR was operated in two stages: Stage 1 for the methanotrophic biofilm formation on HFMs (days 0–147) and Stage 2 for the tests of bioelectrochemical CH_4_ oxidation (days 147–255). During Stage 1, nitrate was used as the electron acceptor and the reactor was operated according to a similar procedure as used for the parent reactor, which has been shown as an effective method for the faster growth of methanotrophic biofilm on HFMs [39–41]. To inoculate the reactor, 100 mL biomass was seeded to the anodic chamber of BEMR, yielding an initial VSS concentration of *ca*. 0.75 g L^−1^. The growing medium consisted of 3 g L^−1^ KH_2_PO_4_, 6 g L^−1^ Na_2_HPO_4_, 0.15 g L^−1^ NH_4_Cl, 0.015 g L^−1^ CaCl_2_·2H_2_O, 0.1 g L^−1^ MgCl_2_·6H_2_O, NaHCO_3_ 1.5 g L^−1^ and trace element [38]. Nitrate and ammonium were introduced to the reactor by manual injection of their stock solutions (2.8 mol/L NO_3_^−^-N and 3.4 mol/L NH_4_^+^-N, respectively), to give initial concentrations between 14.0 to 15.0 mmol N/L for the two chemicals. Samples were taken regularly to monitor nitrate and ammonium consumption, and both nutrients were topped-up to their initial concentrations once their levels dropped below 4.0 mmol N/L. Nitrogen removal rates within each nutrient feeding pulse were determined as the slope of the concentration profiles of nitrate and ammonium. CH_4_ was continuously supplied to the reactor by applying 1.5 bar of pressure to the lumen of the HFM. The successful colonization of biofilm was determined by a gradual stabilization of nitrate removal rate.

During Stage 2, the cathodic chamber was filled with electrolyte containing 50 mM of phosphate buffer solution. Nitrate and ammonium additions were discontinued, the BEMR was connected to a potentiostat (CHI 1030C, CH Instruments Inc, USA), and, under the hypothesis that the anode would act as the final electron acceptor instead of nitrate, the anode electrode was polarized at 0.5 V versus an Ag/AgCl reference electrode in 3 M KCl (MF-2053, Basi, USA, + 0.210 V *vs* the standard hydrogen electrode, SHE). Potassium ferricyanide (K_3_[Fe(CN)_6_]) was added into the anodic chamber at a concentration of 1 mM to enable electron transfer between the biofilm grown onto the HFMs and the anode electrode. CH_4_ supply to the anode chamber was again obtained by pressurizing the HFM with CH_4_ gas. Different pressures (i.e. 0, 0.8, 1.2, 1.6 bar) were applied during this stage to investigate the correlation between CH_4_ availability and current output.

To provide control measurements, an identical of bioelectrochemical system (Control-BES) was set up and was operated under the same conditions to the BEMR, however, without HFMs assembly and amendment of ferricyanide as the redox mediator. CH_4_ in the Control-BES was fed directly to the headspace of the anodic chamber. Performance of CH_4_ oxidation was compared between the BEMR and the Control-BES by monitoring their current output as well as CO_2_ accumulation from AOM in the reactors throughout the operational course. As strong PBS was used in the anodic electrolyte, all of the CO_2_ produced from AOM was trapped in the solution. To measure CO_2_ accumulation in the solution, 0.5 mL of liquid samples was collected from the anode chamber and injected into 3 mL Exetainer vials (Labco, UK) for dissolved CO_2_ quantification.

To further verify the role of ferricyanide in bridging electron transfer from methanotrophic biofilm on HFMs to physically separated anode, a control experiment was performed by comparing chronoamperometric current in the presence and absence of ferricyanide. In the ferricyanide-amended BEMR with stable current output under the condition of pressurizing HFMs at 0.8 bar, ferricyanide was removed from the anolyte by continuous medium change in order not to upset biofilm on the HFMs. Specifically, as show in Additional file 1: Fig. S1, a feeding bottle was connected to the anode chamber, and 1 L of deoxygenated and CH_4_-saturated medium was pumped through the anode chamber into an overflow bottle at a flow rate of 5 mL/min to gradually wash ferricyanide away. A 1 L gas bag filled with CH_4_ was connected to the headspace of the feeding bottle to avoid vacuum during pumping. Liquid from the anode chamber was sampled every 15–30 min to quantify the variation of ferricyanide concentration over time. After the ferricyanide was removed from the anode chamber, a second stage of the test was performed by change the medium in the feeding bottle to 1 L of deoxygenated and CH4-saturated medium containing 1 mM ferricyanide to add it back to the anode chamber. Chronoamperometric current response to ferricyanide removal and addition was recorded at a poised potential of + 0.5 V Ag/AgCl to show the correlation between ferricyanide concentration and current output. Both BEMR and Control-BES were operated in batch mode, with internal mixing obtained by using magnetic bar and a magnetic stirrer set at 200 rpm. Both systems were operated at ambient temperature of 24 ± 1 °C.

### Electrochemistry analysis

Bioelectro-catalytic oxidation of CH_4_ in the BEMR and Control-BES was determined by a chronoamperometry (CA). The current *versus* time traces resulting from the application of a fixed anodic potential of 0.5 V were automatically constructed by the potentiostat with a resolution of 100 s per sample.

In addition, cyclic voltammetry (CV) was performed on the anodes of both of BEMR and Control-BES at the end of the experimentation using a multichannel potentiostat (Potentiostat/Galvanostat VMP3, Biologic Science Instrument, France). CVs traces were recorded between a potential window of − 0.7/ + 0.6 V *vs* Ag/AgCl at the scan rate 1.0 mV s^−1^.

### Analytical methods

Liquid samples were taken from batch incubations and BEMR using sterile syringes and needles and were immediately filtered through 0.22 μm disposable sterile Millipore filter (Merck). The concentrations of nitrogenous compounds (including NH_4_^+^, NO_3_^−^) were measured with a Lachat QuickChem 8000 flow injection analyzer (Lachat Instrument, Milwaukee, WI). K_3_[Fe(CN)_6_] and K_4_[Fe(CN)_6_] were quantified by a spectrophotometric method as detailed in Lai et al. [25]. For the CO_2_ measurement, the samples stored in Exetainer vials were acidified with 0.2 mL anaerobic HCl solution (1 mM) and kept for at least 1 h before GC/MS measurement to allow CO_2_ to equilibrate with the headspace. Quantity and isotopic composition of CO_2_ in the headspace of Exetainer vials was measured by GC/MS (GC 7890A coupled to MSD 5975C; Agilent) as details described in Additional file 1.

### Field-emission scanning electron microscope (FESEM)

FESEM images were obtained using a JOEL-7100F Field Emission Scanning Electron Microscopy. Details of sample preparation are provided in the Supporting Information.

### 16S rRNA gene sequencing

16S rRNA gene sequencing was applied to characterize microbial community dynamics. Total cell DNA was extracted using FastDNA SPIN for Soil kit (MP Biomedicals, USA) according to the manufacturer’s instructions. The extracted DNA concentration was quantified with NanoDrop 2000 (Thermo Fisher Scientific, USA). The 16S rRNA gene was amplified using the universal primer set 926F (5′-AAACTYAAAKGAATTGACGG-3′) and 1392R (5′-ACG-GGCGGTGTGTRC-3′). A QIAquick PCR Purification Kit (Qiagen) and a Quant-iT dsDNA HS assay kit (Invitrogen) were employed to purify and quantify the PCR products, respectively. Amplicons were pooled in equimolar concentration and sequenced with an Illumina sequencer (Illumina, USA). Raw sequencing data were quality-filtered and demultiplexed using Trimmomatic, with poor-quality sequences trimmed and removed. Subsequently, high-quality sequences at 97% similarity were clustered into operational taxonomic units (OTUs) using QIIME with default parameters, and representative OTU sequences were taxonomically aligned against Greengenes 16S rRNA database.

### Calculation of coulombic efficiency

The coulombic efficiency (CE), defined as the fraction of electrons recovered from CH_4_ oxidation, was calculated as:

$$ {\text{CE}} = \frac{{\left( {\int\limits_{0}^{T} {It{\text{d}}t} } \right)}}{(enF)}, $$

where *I *is the current output observed in BESs, *e* is the moles of electrons from each mole of CH_4_ oxidation (8 for CH_4_ from the half reaction: CH_4_ + 2H_2_O → CO_2_ + 8H^+^  + 8e^−^), *n* is the detected CO_2_ production (mol), *F* is Faraday’s constant (96,485 C mol^−1^).

## Supplementary information

**Additional file 1.** GC/MS Instrument method. Samples preparation for SEM. Additional table and figures.

## Data Availability

All data generated or analysed during this study are included in this manuscript and its Additional file 1.

## Abbreviations

ANME: Anaerobic methanotrophic archaea
AOM: Anaerobic oxidation of methane
BEMR: Bioelectrochemical membrane reactor
BESs: Bioelectrochemical systems
CV: Cyclic voltammetry
EET: Extracellular electron transfer
GDE: Gas-diffusion electrode
GTL: Gas-to-liquid
HFMs: Hollow fibre membranes
MCR: Methyl-coenzyme M reductase
SOCFs: Solid fuel cells
VSS: Volatile suspended solids
