# Supplementary material for: Enhancing methane oxidation in a bioelectrochemical membrane reactor using a soluble electron mediator
Source: Biotechnol Biofuels. 2020 Oct 16;13:173. doi: 10.1186/s13068-020-01808-7 (PMC7568384; doi:10.1186/s13068-020-01808-7)
Supplement: Supplementary file 1 — Additional file 1. GC/MS Instrument method. Samples preparation for SEM. Additional table and figures. [file 13068_2020_1808_MOESM1_ESM.docx]

**Additional information for**

**Enhancing methane oxidation in a bioelectrochemical membrane reactor using a soluble electron mediator**

Xueqin Zhang^1^, Hesamoddin Rabiee^1^, Joshua Frank^1^, Chen Cai^1^, Terra Stark^2,3^, Bernardino Virdis^1^, Zhiguo Yuan^1^ and Shihu Hu^1^*

^1^Advanced Water Management Centre, Faculty of Engineering, Architecture and Information Technology, The University of Queensland, 4072 Brisbane, Australia

^2^Australian Institute for Bioengineering and Nanotechnology, The University of Queensland, 4072 Brisbane, Australia;

^3^Queensland Node of Metabolomics Australia, The University of Queensland, 4072 Brisbane, Australia;

**Correspondence:**

[s.hu@awmc.uq.edu.au](mailto:s.hu@awmc.uq.edu.au)

**This file includes on 11 pages:**

GC/MS Instrument method

Samples preparation for scanning electron microscopy (SEM)

Tables S1, S2

Figures S1-S5

**GC/MS Instrument method**

GC/MS – An Agilent 7890A gas chromatograph that was coupled to an Agilent 5975C quadrupole mass spectrometer (Agilent Technologies, Santa Clara, CA) with a Gerstel Autosampler (MPS 2 XL).

[Gas chromatography](https://www.sciencedirect.com/topics/agricultural-and-biological-sciences/gas-chromatography) was performed using a 30 m J & W HP-PLOT Q PT column (Agilent Technologies, Santa Clara, CA) with a 530 μm internal diameter and 40 μm film thickness. Helium was used as carrier gas at a constant gas flow of 5.58 ml/min.

The GC oven temperature was held at 50 °C for 2 min with an injection volume of 250uL in split mode with a split ratio of 25:1.

The mass spectrometer scanned over the range m/z 10–50, maintaining the temperature of the mass detector at 150 °C, the transfer line at 200 °C while the ion source was kept at 230 °C.

CO2 and CH4 detection was attained in positive EI at 70 eV using the standard autotune procedure for mass calibration. Acquisition was performed in Total Ion Chromatography (TIC) for identification and in Selected Ion Monitoring (SIM) for quantitation purpose monitoring m/z signals at 44 and 45 Da (CO2) 16 and 17 Da (CH4) with a dwell time of 30 ms for each signal.

Data processing was performed using the Chemstation program (Agilent Technologies, Santa Clara, CA). The gases were identified by comparison of their mass spectra with those in the NIST 11 library.

**Samples preparation for scanning electron microscopy (SEM)**

A standard microwave-assisted conventional chemical fixation and drying methodology was used for preparing SEM samples. Hollow fibers with biofilm on surface were immediately fixed using a fixative mixture of 2.5% (v/v) glutaraldehyde (electron microscopy grade, ProSciTech, Thuringowa, QLD, Australia) in 100 mM HEPES buffer (pH 7.4) at room temperature under anaerobic conditions. The quantity of fixative used was near a 1:1 volumetric ratio to the sample. Before further preparation, samples were washed with 100 mM HEPES buffer (pH 7.4) two times.

Further processing was done with a microwave processor equipped with a cooler (22 °C; Pelco Biowave® 34700 Microwave with SteadyTemp™ Cooler, Ted Pella Inc., Redding, CA, USA). The dehydration was done in a graded series of ethanol from 20% to 100%, and finally dried in HMDS (2x). The Biowave was set at 150W for 40 seconds under 74.5 kPa vacuum for each step.

The samples were mounted onto aluminium stubs using conductive double-sided adhesive carbon tabs (ProSciTech). The stubs were then sputter coated with platinum (EIKO IB-5 Sputter Coater, EIKO Engineering Co. Ltd., Hitachinaka, Japan) to achieve a uniform layer over the membrane surface.


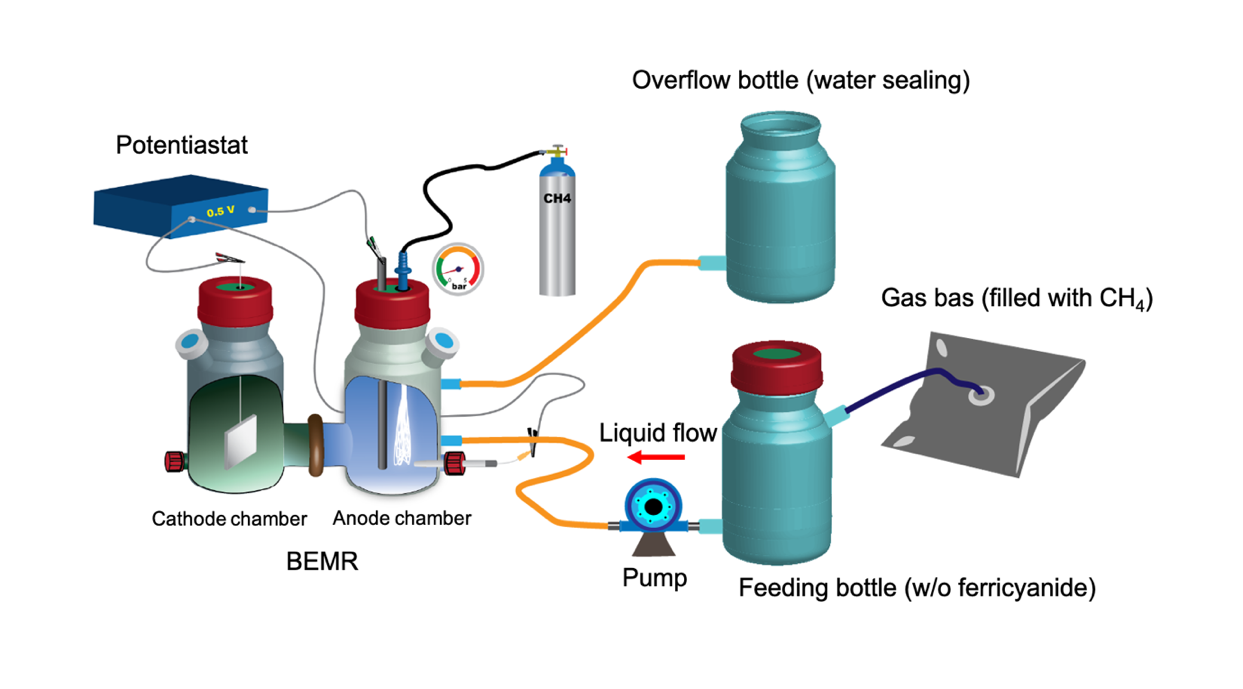


Fig. S1 Schematic diagram of BEMR setup for ferricyanide removal and amendment in a control experiment


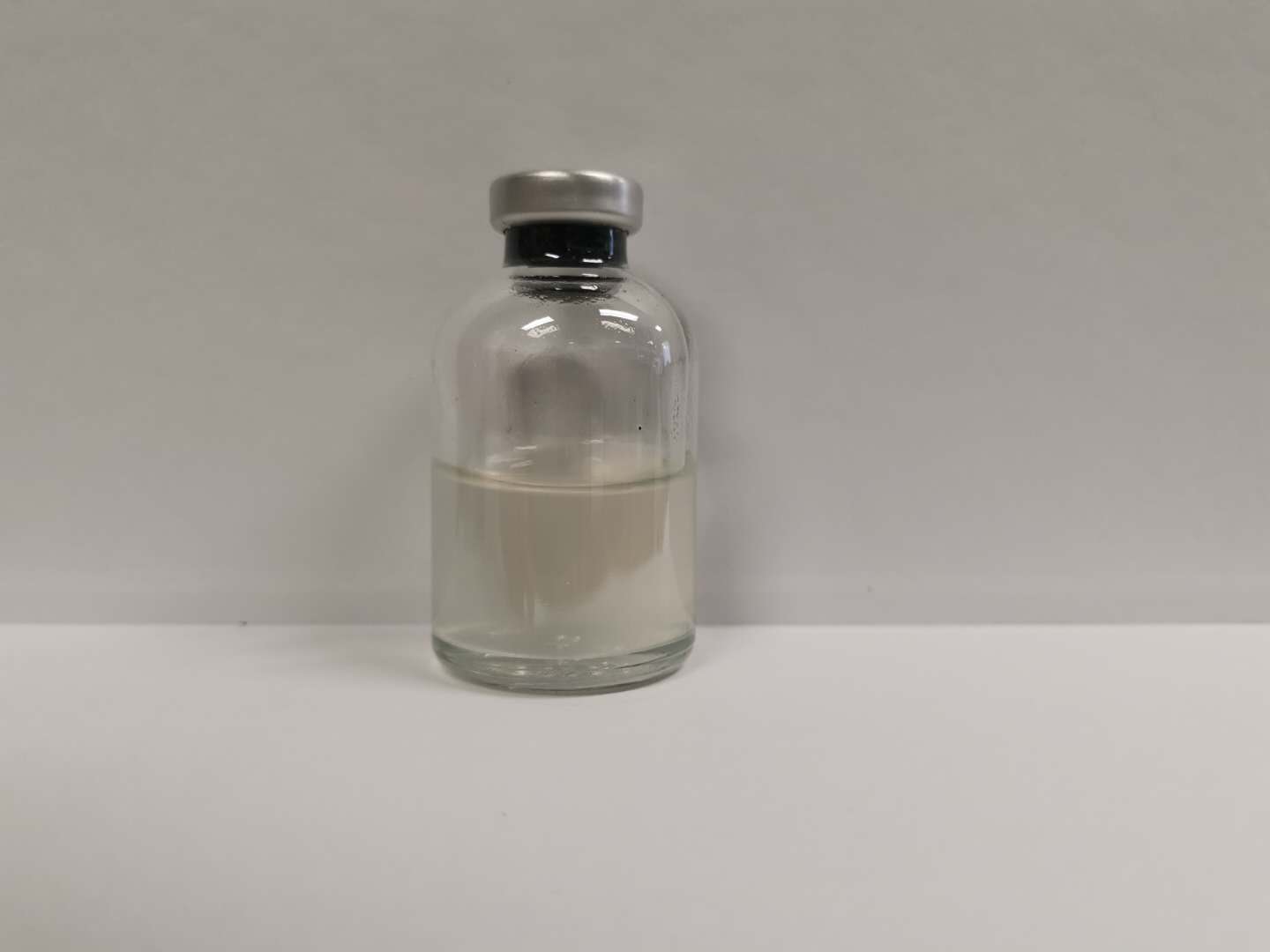

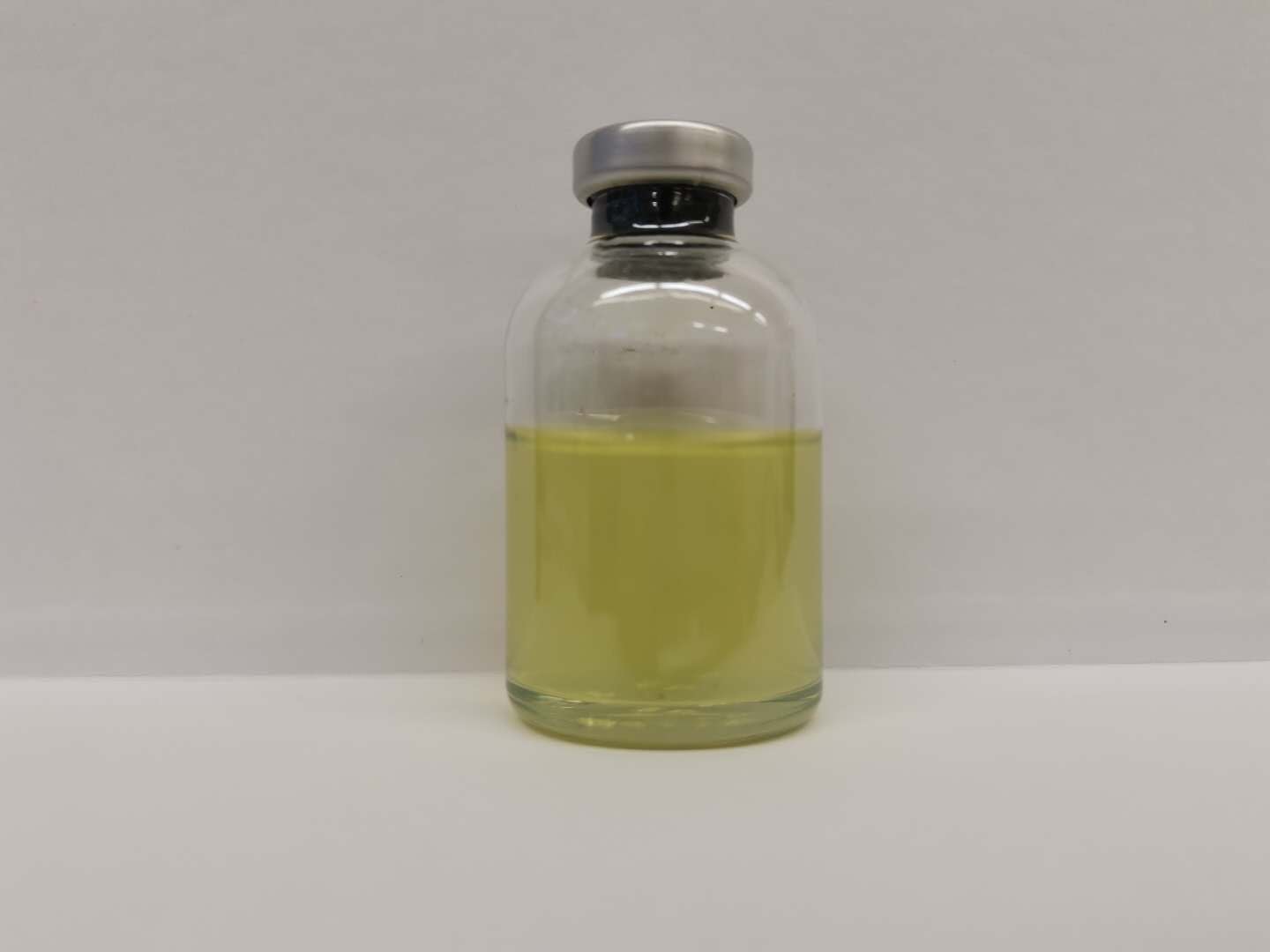


(b)

(a)

Fig. S2 Digital photographs displaying colour change in incubations for ferricyanide reduction by a ANME-dominated mixed culture at, (a) t = 0, (b) t = 48 h. Ferricyanide was supplied at 1 mM, and methane was fed in the headspace.


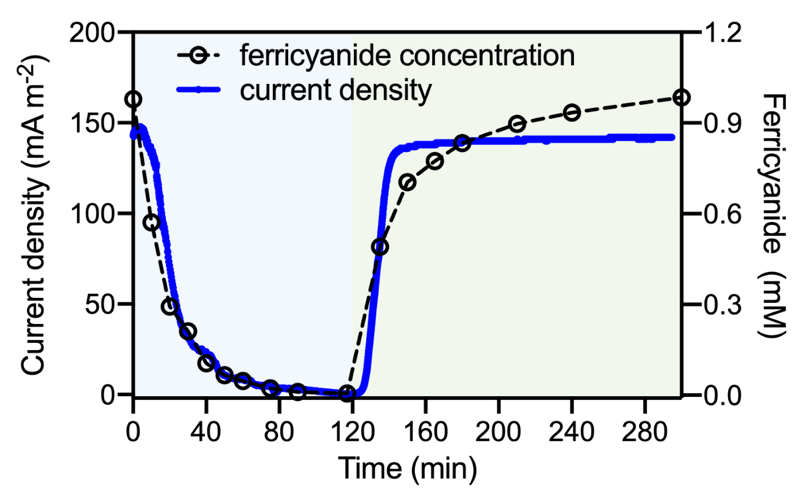


Fig. S3 Chronoamperometric current response to ferricyanide variation in the mediator manipulation experiment


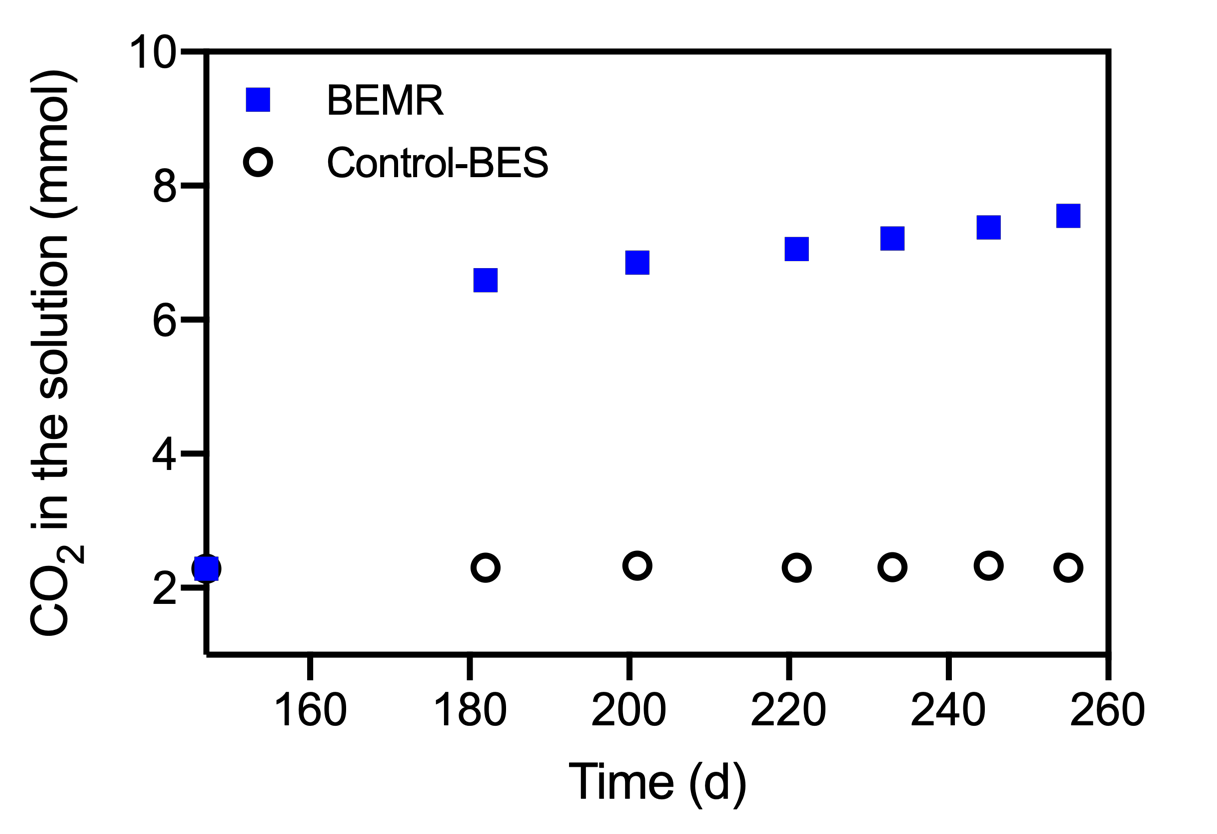


Fig. S4 CO_2_ accumulation towards bioelectrochemical CH_4_ oxidation in the BEMR and the Control-BES


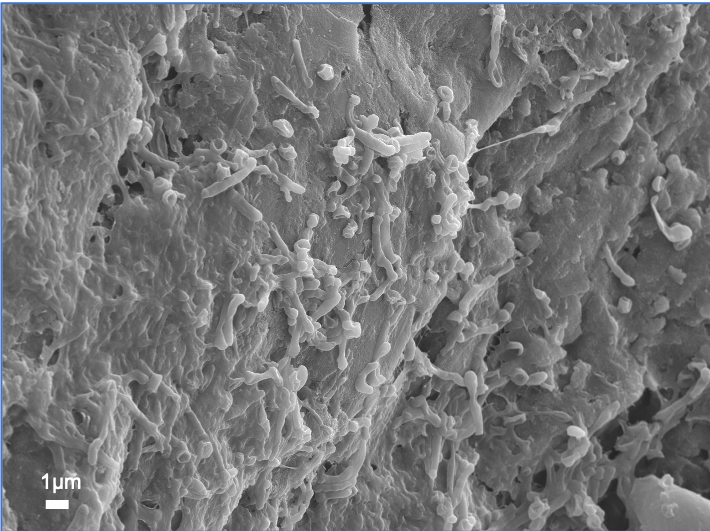


Fig. S5 Scanning electron microscope (SEM) image showing biofilm formed on the surface of graphite anode in the Control-BES on day 255

| Period (day) | Coulombic efficiency (%) |
| --- | --- |
| 147-182 | 54.3 |
| 183-201 | 93.5 |
| 202-221 | 98.1 |
| 222-233 | 92.2 |
| 234-245 | 94.3 |
| 246-255 | 104.3 |
| 183-255 (average) | 96.5 ± 4.9 |

Table S1 Coulombic efficiency of BEMR at different periods

Table S2 Summary of bioelectrochemical methane oxidation in different studies

| Anode materials | CH_4_ feeding strategy | Maximum current density (mA m^–2^) | CH_4_ activator | Proposed EET pathway | Ref |  |
| --- | --- | --- | --- | --- | --- | --- |
| Carbon fiber felt | Soluble CH_4_ dissolved from headspace | 23.8 | ANME | DET or DIET (with *Geobecter*) | [1] | |
| Carbon fibers | Pre-saturated CH_4_ in medium | 11 | *Methanosarcina acetivorans* | Intermediate-dependent IET (*Geobacter* as partner) | [2] | |
| Carbon brush | Soluble CH_4_ dissolved from headspace | 273 | Engineered *M. acetivorans* | Acetate-dependent IET (*Geobacter* as partner) | [3] | |
| Carbon cloth | CH_4_ diffusion by GORE-TEX cloth | 1130.2 | *Methanobacterium* | Acetate-dependent IET (*Geobacter* as partner) | [4] | |
| Carbon brush | Soluble CH_4_ dissolved from headspace | 7300 | Engineered *M. acetivorans* | Acetate-dependent IET (Shuttled by mediators ) | [5] | |
| Hollow fibres + carbon brush | CH_4_ diffusion by hollow fibres | 196 | ANME | DET | This study | |

DET: direct electron transfer; IET: Interspecies electron transfer

**References**

1. Ding J, Lu Y-Z, Fu L, Ding Z-W, Mu Y, Cheng SH, Zeng RJ: Decoupling of DAMO archaea from DAMO bacteria in a methane-driven microbial fuel cell. Water research 2017, 110:112-119.

2. Gao Y, Lee J, Neufeld JD, Park J, Rittmann BE, Lee H-S: Anaerobic oxidation of methane coupled with extracellular electron transfer to electrodes. Scientific reports 2017, 7(1):1-9.

3. McAnulty MJ, Poosarla VG, Kim K-Y, Jasso-Chávez R, Logan BE, Wood TK: Electricity from methane by reversing methanogenesis. Nature communications 2017, 8(1):1-8.

4. Yamasaki R, Maeda T, Wood TK: Electron carriers increase electricity production in methane microbial fuel cells that reverse methanogenesis. Biotechnology for biofuels 2018, 11(1):211.

5. Yu L, Yang Z, He Q, Zeng RJ, Bai Y, Zhou S: Novel Gas Diffusion Cloth Bioanodes for High-Performance Methane-Powered Microbial Fuel Cells. Environmental science & technology 2018, 53(1):530-538.
